# Robert H. Norgate

**Published:** 1925-01

**Authors:** 


### ROBERT H. NORGATE, M.R.C.S., L.R.C.P.

The death of Robert H. Norgate, which occurred at Southmead Hospital on December 10th, has removed a striking personality from our midst. He was born at Shirehampton in 1865, the family removing a little later to Bristol, where he received his earlier education at the Grammar School.

In course of time he joined the Bristol Medical School, doing most of his clinical work at the Royal Infirmary, and obtaining the diplomas of M.R.C.S., L.R.C.P. in 1891.

For a short time he was engaged in private practice, and was Medical Officer to the Bristol Foresters for two years. He also acted as assistant medical officer in mental hospitals at Worcester and Maidstone, at which institutions he laid the foundation of his wide knowledge of psychiatry, which proved of great value to him in later years.

The greater part of his professional life was given to the service of the Bristol Board of Guardians, first at the Stapleton and Eastville Institutions, and more recently at Southmead Hospital, where he was Medical Superintendent at the time of his decease.

The period of his public service extended over nearly thirty years, during which he displayed unceasing zeal in the execution of his many duties.

It has been truly said that the real success of a man's life is not measured by the honours that he obtains for himself but by what he does for others, and therefore Robert Norgate will rank for all time as one of Bristol's most valued men. Throughout his professional career he invariably took a friendly interest in each individual coming under his care; he was not only acquainted with the medical histories of his patients—often extending over long periods—but he also spared no pains to gain information about their personal interests, and was thus able to give them helpful advice in times of trouble; this striking characteristic was exhibited up to the very end, for within a few hours of his death he was inquiring after certain of the patients in hospital. It was this happy gift which endeared him to the thousands of people who came in contact with him during his work.

Robert Norgate was also greatly esteemed by all those who were associated with him at Southmead; his medical colleagues had a very high opinion of his clinical acumen, and the nurses greatly appreciated his lectures, which were notable for their clearness.

An idea is prevalent among a certain section of the community that research can only be carried out by those who devote their whole time to laboratory work. Norgate proved the fallacy of such an idea, and did much to show that clinical research is at least as useful as that done in the laboratory. One of his most important observations was on the use of pituitrin in the control of certain types of hemorrhage and in the alleviation of distress in malignant disease; he also did excellent work on the value of injections of sterilised olive oil in selected cases of arthritis and in trigeminal neuralgia. The chief object of all his investigations was to relieve suffering, and not to build up fame for himself.

Keenly interested in all forms of sport, especially Rugby football, the familiar figure of Robert Norgate will be sadly missed at matches, where he was a frequent spectator long after he ceased to play himself; he was also passionately fond of anything connected with shipping, and spent much of his leisure time in making water-colour paintings of ships.

The death of such an excellent clinician is a great loss to the medical profession, but a far greater loss to the large army of unfortunate individuals who have experienced more sorrow than joy in their journey through life.

His widow and daughter are left to mourn his loss, but will be comforted by the knowledge that the results of his labours will remain with us always.

